# The impact of prevalent primary cancers in melanoma patients on survival

**DOI:** 10.3389/fonc.2026.1854567

**Published:** 2026-09-14

**Authors:** Alessandra Buja, Stefano Guzzinati, Chiara Trevisiol, Claudio Palmeri, Marcello Di Pumpo, Paolo Del Fiore, Carlo Riccardo Rossi, Carmen Stocco, Pierfranco Conte, Manuel Zorzi, Simone Mocellin

**Affiliations:** 1 Department of Cardiac, Thoracic, Vascular Sciences, and Public Health, University of Padova, Padua, Italy; 2 Veneto Tumour Registry (RTV), Azienda Zero, Padua, Italy; 3 Soft-Tissue, Peritoneum, and Melanoma Surgical Oncology Unit, Veneto Institute of Oncology IOV- IRCCS, Padua, Italy; 4 Euganea Local Health Authority, AULSS6 Veneto Region, Padua, Italy; 5 Department of Surgery, Oncology, and Gastroenterology - DISCOG, University of Padova, Padua, Italy; 6 San Camillo IRCCS Hospital, Venice, Italy; 7 Periplo Foundation, Cremona, Italy

**Keywords:** cohort study, comorbidity, cutaneous malignant melanoma, health care costs, registries, survival

## Abstract

**Background:**

The presence of prior malignancies at the time of cutaneous malignant melanoma diagnosis may be a critical determinant of patient survival. The present study evaluated the clinical and economic impact of prevalent cancers at the time of cutaneous melanoma diagnosis.

**Methods:**

This population-based retrospective cohort study included melanoma cases identified in the Italian Veneto Region Cancer Registry (RTV) in 2019 and 2021. Prevalent primary cancers that occurred within a five-year interval prior to melanoma diagnosis were identified from the RTV’s multiple tumors dataset. Kaplan–Meier and Cox proportional hazards models were used to test the association between prevalent primary cancers and overall and melanoma-specific survival. Over three years, healthcare costs were analyzed from a health system perspective using administrative data.

**Results:**

Out of 2,978 melanoma patients, 134 (4.5%) had prevalent tumors. The most frequent additional cancer sites were prostate (30.2%), breast (10.8%), colon (7.9%), and kidney (7.2%). Prevalent malignancies were associated with older age, male sex, and greater comorbidity burden in melanoma patients. In univariate Cox regression analysis, prevalent tumors were associated with an increased risk of all-cause mortality (HR 2.23, 95% CI 1.59–3.15; p<0.001), whereas no significant association was observed with CM-specific mortality. After adjustment for prognostic factors, prevalent tumors remained independently associated with an increased risk of all-cause mortality (aHR 1.72, 95% CI 1.19–2.47; p=0.004), while the association with CM-specific mortality remained non-significant. Overall and melanoma-related costs were higher in melanoma patients with prevalent tumors (overall three-year average costs € 30,698.34 vs. € 17,471.23).

**Conclusions:**

The presence of prevalent tumors at the time of melanoma diagnosis influences the direct costs associated with healthcare. These findings highlight the significance of integrating multimorbidity considerations into the management and resource allocation in patients with cutaneous melanoma.

## 1 Introduction

Cutaneous malignant melanoma (CM) represents a significant and growing global public health challenge. It is responsible for the vast majority of skin cancer-related mortality. Globally, an estimated 330,000 new cases of melanoma were diagnosed in 2022, resulting in approximately 60,000 deaths worldwide (1). Data from the Global Burden of Disease (GBD) study highlight that between 1990 and 2021, the global prevalence of CM grew by 161%, reflecting rising exposure to ultraviolet (UV) radiation, improved detection practices, and an aging population (2). Despite the sharp increase in incidence, mortality trends have been favorable in many high-income countries (3).

Recent population-based evidence from the Veneto Region Cancer Registry (4) found that CM patients with a history of multiple chronic diseases face an up to threefold increase in overall mortality risk (HR 2.67 to 3.08) compared to those without this history. Also, prevalent malignancies existing at the time of CM diagnosis could be a critical determinant of CM patients’ survival. The presence of an antecedent malignancy may, in fact, complicate the clinical trajectory of melanoma. A study by Zhou et al. shows how melanoma patients with a prior cancer history face a 23% increased risk of all-cause mortality (HR = 1.23) compared to those affected only by melanoma, suggesting that this factor is a critical determinant of overall survival (5). This is driven by clinical, biological, and genetic factors. Diagnostic interference and “stage migration” are key contributors, supported by data from the Swedish Melanoma Register (6) showing that pre-existing health burdens often lead to the detection of thicker, more advanced melanomas (e.g., Stage IIC), suggesting that intensive surveillance for a prior cancer may overshadow the early signs of a new primary tumor. In addition, previous oncological treatments may both impair the immune system and limit melanoma therapeutic options in order to avoid toxicity or medication overload.

Further evidence is needed to inform tailored management strategies for this oncological disease. The present study, therefore, aims to analyze the impact of prevalent cancers at the time of CM diagnosis on the survival and direct costs of CM patients, using population-based data from the Italian Veneto Region.

## 2 Materials and methods

This population-based retrospective cohort study included 2,978 patients diagnosed with cutaneous melanoma. CM cases were identified from the 2019 and 2021 records of the Veneto Region Cancer Registry (RTV). These calendar years were selected because high-resolution melanoma-specific data were available in the registry (7). RTV is a certified population-based cancer registry that systematically records all malignant neoplasms diagnosed among residents of the Veneto Region, which has a population of approximately 4.9 million inhabitants (8). Case ascertainment is based on an integrated information system that includes pathology reports, providing Breslow thickness, ulceration status, tumor histology, pT, pN, and M categories and the resulting AJCC 8th edition pTNM stage (9) as well as clinical records, death certificates, and administrative public health databases. Vital status and cause-of-death information were obtained through linkage with the regional mortality registry, which also captures deaths occurring outside the regional territory (10).

The prevalent primary cancers, characterized as separate malignancies diagnosed independently of CM and existing at the time of CM diagnosis—occurring within a five-year interval prior to CM diagnosis—were identified from a specialized RTV’s dataset that records additional tumor diagnoses. Only prevalent primary cancers were considered, patients with second primary cancers diagnosed after the CM diagnosis were classified as not having additional tumors.

### 2.1 Statistical analysis

Descriptive statistics were used to summarize patient, tumor, and clinical characteristics.

Survival outcomes were estimated using the Kaplan–Meier method, and differences between groups were assessed with the log-rank test. Three-year overall survival and CM-specific survival were calculated for the entire cohort. Stage-stratified survival analyses were performed by combining pTNM stages I and II into an early-stage group and pTNM stages III and IV into a late-stage group to improve the robustness of the estimates given the limited number of patients with prevalent tumors.

Multivariable Cox proportional hazards regression models were then constructed to estimate adjusted hazard ratios (HRs) with 95% confidence intervals (CIs) for the association between the presence of concurrent non-melanoma cancers at CM diagnosis and survival outcomes. Separate models were fitted for overall mortality and CM-specific mortality, adjusting for sex, age at diagnosis, and TNM stage. Proportional hazards assumptions were tested for each covariate using Schoenfeld residuals. Missing data were handled using complete-case analysis.

### 2.2 Cost analysis

The cost analysis was evaluated from a health system perspective, considering only the direct costs incurred by the regional healthcare service and conducted using anonymized aggregate data. The cost estimates encompass a 3-year period following the initial cancer diagnosis.

Each patient was assigned a unique and anonymous identification code, which was used to link all administrative data covering hospital admissions, drug prescriptions, outpatient visits, emergency room visits, hospice admissions, medical devices, and vital status information.

To identify melanoma-specific costs, the most frequent diagnostic, procedural, pharmaceutical, and healthcare service codes (defined as those with a prevalence >1% in the study cohort) were extracted from each respective administrative dataset, manually reviewed, and classified as either melanoma-specific or not melanoma-specific.

Daily costs per patient alive and average costs in the 3 years following the diagnosis were calculated and stratified by stage at diagnosis. Daily costs were estimated using a weighted average based on the number of days each patient was alive during the 3-year follow-up period.

Both overall and CM-specific costs were computed. The distribution of both cost variables was assessed for normality using the Shapiro–Wilk test, performed separately for patients with and without prevalent tumor diagnosis, both overall and within each disease stage. As the normality assumption was not satisfied, differences in costs according to prevalent tumor status were assessed using the Kruskal–Wallis test. Tobit regression models, left-censored at zero, were used to examine the impact of the presence of a prevalent tumor on both overall and CM-specific healthcare three-year costs, while adjusting for sex, age at diagnosis, and TNM stage. The Tobit model fits for continuous responses where the outcome variable is censored at zero value. These models were performed using complete-case analysis, including only patients with complete information for all variables.

Statistical significance was defined as a two-sided p-value < 0.05. All data management and statistical analyses were performed using R version 4.3.2.

### 2.3 Ethics statement

This retrospective study involving human participants was conducted in accordance with the ethical standards of the institutional research committee and with the 1964 Helsinki Declaration and its later amendments or comparable ethical standards. This study project was formally approved by the Ethics Committee of the Veneto Oncological Institute (protocol number 52/2016). All data were anonymized in accordance with Italian regulations and used for monitoring and quality assurance purposes. The data analyses were performed on anonymous, aggregated data, ensuring that no individual could be identified.

## 3 Results

Among 2,978 melanoma patients, 134 individuals accounted for 139 multiple tumors; most (96.3%) had one additional malignancy, while 3.7% had two. The median time between the diagnosis of prior primary tumors and CM was 813 days, ranging from 275.5 to 1260 (mean: 818.6 days, SD: 553.4). The most frequent additional cancer sites were prostate (30.2%), breast (10.8%), colon (7.9%), kidney (7.2%), bladder (5.8%), and thyroid (3.6%) (**Table 1**).

**Table 1:** Frequency distribution of prevalent tumors.

Variable	% (Value)
N° additional tumors found in melanoma incident cases	
0	95.5% (2844)
1	4.3% (129)
2	0.2% (5)
TOTAL	100% (2978)
Prevalent tumors ICD-10 code (first three characters)	
Malignant neoplasm of prostate [C61]	30.2% (42)
Malignant neoplasm of breast [C50]	10.8% (15)
Malignant neoplasm of colon [C18]	7.9% (11)
Malignant neoplasm of kidney, except renal pelvis [C64]	7.2% (10)
Malignant neoplasm of bladder [C67]	5.8% (8)
Malignant neoplasm of thyroid gland [C73]	3.6% (5)
Malignant neoplasm of stomach [C16]	2.9% (4)
Malignant neoplasm of bronchus and lung [C34]	2.9% (4)
Malignant neoplasm of corpus uteri [C54]	2.9% (4)
Malignant neoplasm of small intestine [C17]	2.2% (3)
Malignant neoplasm of rectum [C20]	2.2% (3)
Malignant neoplasm of pancreas [C25]	2.2% (3)
Non-follicular lymphoma [C83]	2.2% (3)
Mature T/NK-cell lymphomas [C84]	2.2% (3)
Multiple myeloma and malignant plasma cell neoplasms [C90]	2.2% (3)
Carcinoma *in situ* of other and unspecified sites [D09]	2.2% (3)
Other	10.8% (15)
TOTAL	100% (139)

Patients with prevalent tumors were more frequently male (71.6% vs 56.8%, *p* = 0.001) and older (median age 73.5 [IQR 65–81] vs 62.0 [IQR 51–74] years, *p* < 0.001). No significant differences were observed between groups for TNM stage (*p* = 0.180), Breslow thickness (*p* = 0.232), ulceration (*p* = 0.460), histological subtype (*p* = 0.491) or primary site (*p* = 0.617) (**Table 2**).

**Table 2:** Sociodemographic and clinical melanoma characteristics by prevalent tumor status.

Variable	Overall dataset (n = 2978)	Prevalent tumors		p-value
		Absent (n = 2844)	Present (n = 134)	
Sex				0.001^a^
Female	42.5% (1267)	1229 (43.2%)	38 (28.4%)	
Male	57.5% (1711)	1615 (56.8%)	96 (71.6%)	
Age				<0.001^b^
Median[IQR]	63.0 [51.0;75.0]	62.0 [51.0;74.0]	73.5 [65.0;81.0]	
Mean(std)	62.6 (15.4)	62.2 (15.4)	71.7 (11.2)	
Age groups				<0.001^a^
<45	12.7% (377)	375 (13.2%)	2 (1.5%)	
45-54	19.0% (567)	560 (19.7%)	7 (5.2%)	
55-64	20.9% (623)	600 (21.1%)	23 (17.2%)	
65-74	22.1% (657)	619 (21.8%)	38 (28.4%)	
75-84	18.8% (560)	509 (17.9%)	51 (38.1%)	
>84	6.5% (194)	181 (6.4%)	13 (9.7%)	
Breslow				0.232^a^
< 0.76 mm	55.5% (1564)	1505 (55.8%)	59 (48.0%)	
0.76 – 1.75 mm	21.6% (608)	577 (21.4%)	31 (25.2%)	
> 1.75 mm	23.0% (648)	615 (22.8%)	33 (26.8%)	
Missing	158	147	11	
Numerical Breslow				0.095^b^
Median[IQR]	0.7 [0.4;1.6]	0.6 [0.4;1.6]	0.8 [0.4;2.0]	
Mean(std)	1.7 (3.0)	1.7 (3.0)	1.8 (2.3)	
Missing	158	147	11	
Ulcerations				0.460^a^
Absent	80.2% (2265)	2168 (80.3%)	97 (77.6%)	
Present	19.8% (560)	532 (19.7%)	28 (22.4%)	
Missing	153	144	9	
T				0.074^c^
T0	1.3% (38)	33 (1.2%)	5 (3.9%)	
T1	64.8% (1874)	1800 (65.2%)	74 (57.4%)	
T2	13.1% (379)	361 (13.1%)	18 (14.0%)	
T3	10.6% (306)	289 (10.5%)	17 (13.2%)	
T4	10.1% (293)	278 (10.1%)	15 (11.6%)	
Missing	88	83	5	
N				0.294^c^
N0	88.4% (2426)	2328 (88.7%)	98 (83.8%)	
N1	7.4% (202)	190 (7.2%)	12 (10.3%)	
N2	2.9% (79)	74 (2.8%)	5 (4.3%)	
N3	1.3% (36)	34 (1.3%)	2 (1.7%)	
Missing	235	218	17	
M				0.521^c^
M0	95.7% (2733)	2611 (95.7%)	122 (94.6%)	
M1	4.3% (123)	116 (4.3%)	7 (5.4%)	
Missing	122	117	5	
TNM stage				0.180^a^
I	72.5% (2080)	1996 (72.9%)	84 (64.1%)	
II	13.8% (396)	372 (13.6%)	24 (18.3%)	
III	9.3% (267)	251 (9.2%)	16 (12.2%)	
IV	4.4% (126)	119 (4.3%)	7 (5.3%)	
Missing	109	106	3	
Tumor’s histologic type				0.491^c^
Superficial spreading melanoma	53.8% (1596)	1530 (54.0%)	66 (50.0%)	
Malignant melanoma	29.0% (861)	823 (29.1%)	38 (28.8%)	
Nodular melanoma	11.2% (333)	314 (11.1%)	19 (14.4%)	
Lentigo maligna	2.3% (69)	67 (2.4%)	2 (1.5%)	
Acral-lentiginous melanoma	1.2% (35)	32 (1.1%)	3 (2.3%)	
Spitzoid melanoma	0.9% (26)	25 (0.9%)	1 (0.8%)	
Melanoma arising from blue naevus	0.8% (25)	24 (0.8%)	1 (0.8%)	
Desmoplastic melanoma	0.7% (20)	18 (0.6%)	2 (1.5%)	
Missing	13	11	2	
Tumor’s site				0.617^c^
Trunk	48.7% (1420)	1359 (48.8%)	61 (48.4%)	
Lower limbs	21.9% (639)	615 (22.1%)	24 (19.0%)	
Upper limbs	16.7% (486)	460 (16.5%)	26 (20.6%)	
Head and neck	12.6% (368)	353 (12.7%)	15 (11.9%)	
Missing	65	57	8	

^a^Pearson’s Chi-squared test, ^b^Mann-Whitney test, ^c^Fisher’s Exact Test for Count Data.

The survival curves are reported as **Figure 1**. At 3 years (**Table 3**), overall survival (OS) for the whole cohort was 86.6% (95%CI 85.3–87.9), and CM-specific survival (MSS) was 93.2% (95%CI 92.2–94.2). OS was significantly lower in patients with multiple tumors compared with those without (74.3% vs 87.2%, *p* < 0.001), whereas MSS did not differ (90.6% vs 93.3%, *p* = 0.497).

**Figure 1:**
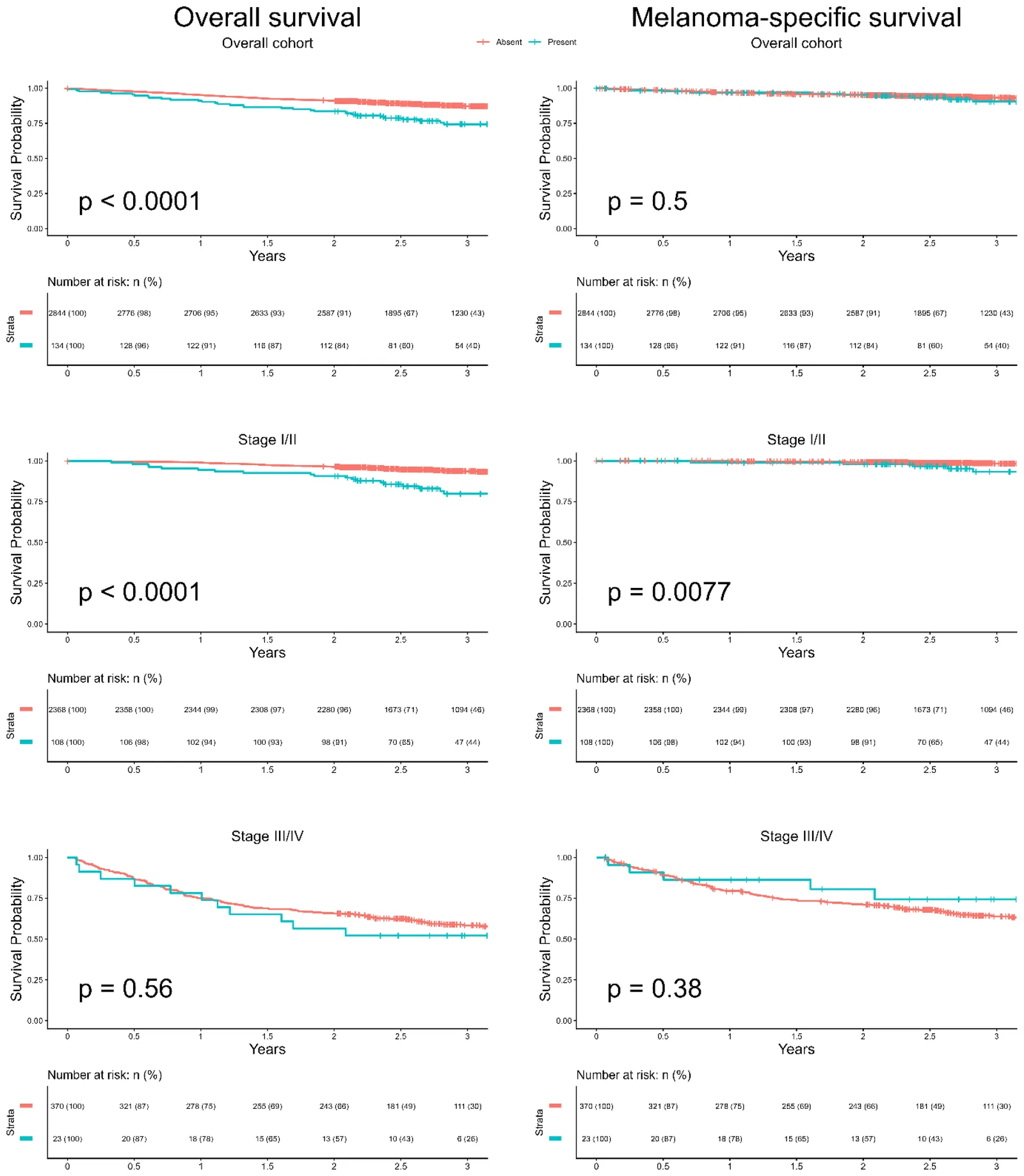
Overall and melanoma-specific mortality by tumor prevalence status and stage at diagnosis.

**Table 3:** Probability of survival three years subsequent to diagnosis, by prevalent tumor status and stratified by melanoma stage at the time of diagnosis.

	Overall survival		Melanoma-specific survival	
Variable	3-year survival [95% CI]	p-value ^a^	3-year survival [95% CI]	p-value ^a^
All patients (n = 2978)	86.6% [85.3% - 87.9%]	–	93.2% [92.2% - 94.2%]	–
Prevalent tumors		<0.001		0.497
Absent	87.2% [85.9% - 88.5%]		93.3% [92.3% - 94.3%]	
Present	74.3% [66.8% - 82.6%]		90.6% [85.0% - 96.5%]	
Stage I and II patients (n = 2476)				
Prevalent tumors		<0.001		0.008
Absent	93.6% [92.5% - 94.7%]		98.4% [97.8% - 99.0%]	
Present	79.9% [72.0% - 88.7%]		93.4% [87.7% - 99.4%]	
Stage III and IV patients (n = 393)				
Prevalent tumors		0.556		0.377
Absent	58.4% [53.3% - 63.9%]		63.9% [58.8% - 69.4%]	
Present	52.2% [35.3% - 77.2%]		74.4% [57.1% - 97.0%]	

^a^log-rank test.

In univariate Cox regression (**Table 4**) analysis, prevalent tumors were associated with increased all-cause mortality (HR 2.23, 95%CI 1.59–3.15; *p* < 0.001), but not with CM-specific mortality (HR 1.25, 95% CI 0.66–2.36; *p* = 0.498). After adjustment for sex, age, and TNM stage, prevalent tumors remained independently associated with worse OS (adjusted HR 1.72, 95% CI 1.19–2.47; p=0.004), whereas no significant association with worse MSS was observed (adjusted HR 1.23, 95% CI 0.64–2.38; *p* = 0.530).

**Table 4:** Cox regression univariate and multivariable analyses.

	Overall survival		Melanoma-specific survival	
Variable	Hazard ratio [95% CI]	p-value	Hazard ratio [95% CI]	p-value
Unadjusted				
Prevalent tumors				
Absent	1.00 [ref]	–	1.00 [ref]	–
Present	2.23 [1.59 – 3.15]	<0.001	1.25 [0.66 – 2.36]	0.498
Adjusted ^a^				
Prevalent tumors				
Absent	1.00 [ref]	–	1.00 [ref]	–
Present	1.72 [1.19 - 2.47]	0.004	1.23 [0.64 - 2.38]	0.530

^a^Adjusted for sex, age, and TNM stage.

**Table 5** reports the average healthcare costs over the three years following CM diagnosis, stratified by stage and by the presence of prevalent tumors. Both overall and CM-specific costs were significantly higher for patients with prevalent tumors. Patients with prevalent cancer face an average daily cost that is almost double those of the others (€ 32.25 and € 17.09, respectively, for overall cost; € 20.52 and €12.83, respectively, for CM-specific cost). The average cost over three years after diagnosis was also significantly higher for CM patients with prevalent tumors: € 30,698.34 and € 17,471.23 (overall cost), and € 19,535.68 and € 13,114.31 (CM-specific cost), respectively, for patients with and without a prevalent malignancy.

**Table 5:** Three-year overall and melanoma specific healthcare costs stratified by stage and presence of prevalent tumors.

		Average daily cost alive patient				Average cost over 3 years after diagnosis			
Dataset		Without prevalent tumors	With prevalent tumors	TOTAL	p-value ^a^	Without prevalent tumors	With prevalent tumors	TOTAL	p-value ^a^
All patients (n=2978)	Overall	17.09	32.25	17.73	<0.001	17,471.23	30,698.34	18,066.41	<0.001
	CM-specific	12.83	20.52	13.15	<0.001	13,114.31	19,535.68	13,403.25	<0.001
Stage I (n=2080)	Overall	8.07	24.22	8.7	<0.001	8,698.08	25,161.31	9,362.94	<0.001
	CM-specific	4.84	13.09	5.16	<0.001	5,217.69	13,603.72	5,556.36	<0.001
Stage II (n=396)	Overall	24.4	36.82	25.08	0.013	24,676.67	33,376.11	25,203.91	0.058
	CM-specific	18.85	23.28	19.1	0.239	19,065.74	21,107.9	19,189.51	0.538
Stage III (n=267)	Overall	65.67	67.57	65.78	0.667	64,719.4	64,751.67	64,721.33	0.674
	CM-specific	57.77	57.46	57.75	0.756	56,937.99	55,062.84	56,825.63	0.995
Stage IV (n=126)	Overall	104.01	66.18	102.81	0.643	42,819.87	15,231.01	41,287.15	0.181
	CM-specific	83.41	24.67	81.54	0.038	34,336.56	5,678.16	32,744.43	0.060
Missing (n=109)	Overall	21.94	35.71	22.2	0.985	17,046.81	18,785.66	17,094.67	0.912
	CM-specific	17.15	30.24	17.4	0.760	13,326.63	15,908.54	13,397.69	0.760

^a^Kruskal–Wallis test. CM, cutaneous melanoma.

The Tobit multivariate regression analysis, performed to evaluate the determinants of three-year costs from diagnosis, confirmed higher costs in patients with prevalent tumors (**Table 6**). The presence of multiple tumors was independently associated with higher overall costs (estimate 9.25, 95% CI 4.92–13.58; *p* < 0.001) and CM-specific costs (estimate 3.90, 95% CI 0.22–7.59; *p* = 0.038).

**Table 6:** Tobit multivariate regression analysis: average three-year costs from diagnosis (dependent variable).

Variable	Overall costs		Melanoma-specific costs	
	Estimate ^a^ [95% CI]	p-value	Estimate ^a^ [95% CI]	p-value
Prevalent tumors				
No	ref	–	ref	–
Yes	9.25 [4.92, 13.58]	<0.001	3.90 [0.22, 7.59]	0.038
Sex				
F	ref	–	ref	–
M	1.40 [-0.44, 3.24]	0.137	0.52 [-1.05, 2.09]	0.515
Age				
<45	ref	–	ref	–
45-54	-0.20 [-3.41, 3.00]	0.901	0.33 [-2.40, 3.06]	0.813
55-64	0.12 [-3.04, 3.28]	0.940	-0.07 [-2.76, 2.62]	0.960
65-74	5.59 [2.43, 8.74]	<0.001	2.80 [0.12, 5.49]	0.041
75-84	5.31 [2.01, 8.60]	0.002	0.19 [-2.62, 3.00]	0.896
>84	-2.85 [-7.59, 1.89]	0.238	-6.09 [-10.13, -2.06]	0.003
Stage				
I	ref	–	ref	–
II	14.91 [12.23, 17.59]	<0.001	13.96 [11.68, 16.24]	<0.001
III	54.58 [51.45, 57.70]	<0.001	51.06 [48.40, 53.71]	<0.001
IV	30.89 [26.45, 35.33]	<0.001	27.47 [23.69, 31.26]	<0.001

^a^Expressed in thousands of euros (€000).

## 4 Discussion

This population-based study evaluated the clinical and economic impact of prevalent primary cancers at the time of melanoma diagnosis. The findings indicate that concurrent malignancies are relatively frequent among CM patients and are associated with older age, and male predominance, while CM pathological characteristics remain broadly comparable to those observed in patients without additional tumors.

The spectrum of previous malignancies — primarily prostate, breast, colorectal and kidney—highlights the importance of integrated survivorship programs and personalized follow-up strategies for cancer survivors. Such an approach should involve harmonizing skin cancer surveillance according to age and sex, alongside meticulous management and monitoring of other malignancies.

Survival analyses demonstrated significantly worse overall survival among patients with prevalent tumors in both univariate and multivariable analyses, whereas CM-specific survival did not differ significantly in either analysis. Different patterns have been reported in population-based analyses of CM occurring in the context of other malignancies, where the impact of prevalent tumors is more pronounced for overall survival than for CM-specific outcomes (11). Moreover, a comprehensive review by Halmágyi et al. showed that CM arising in patients with chronic lymphocytic leukemia (CLL) is associated with markedly worse outcomes, attributed in part to immune dysfunction and treatment constraints (12). A review by Royle et al. reports substantially increased CM mortality in patients with CLL (13), with higher mortality odds compared with CM patients without CLL, and significantly increased risks of CM-specific mortality and recurrence. The poorer overall survival may be attributed to shared etiological pathways that predispose individuals to adverse outcomes. Genetic susceptibility, chronic inflammation, environmental exposures, and systemic immune dysregulation have all been implicated in the development not only of CM and other cancers but also of other acute and chronic pathologies (14–16). Furthermore, the combined effect of pre-existing cancer and comorbidities associated with cancer therapy can result in diminished physiological reserve, increased vulnerability to acute events, and heightened susceptibility to non-melanoma mortality (17). From a clinical perspective, these results highlight the need for tailored management strategies for CM patients with prevalent tumors. These individuals represent a particularly vulnerable subgroup who may benefit from coordinated multidisciplinary care and careful balancing of treatment risks and benefits.

From a health-economic perspective, the presence of prevalent tumors was associated with significantly higher three-year overall and CM-related costs, even after controlling for demographic and clinical variables. The overall-costs difference may largely hinge on how concurrent malignancy is handled. However, this increase was largely driven by patients with stage I melanoma, mainly in melanoma-specific costs. The markedly higher costs observed among patients with stage I melanoma and a previous cancer history likely reflect that clinicians may adopt a more cautious management strategy in patients with previous malignancies, resulting in increased healthcare utilization even when melanoma is diagnosed at an early stage. The finding that melanoma-specific costs were also significantly higher suggests that previous cancer history influences not only general medical care but also the diagnostic and therapeutic pathway directly related to melanoma. Conversely, the absence of significant cost differences among patients with stage II and III disease suggests that, once melanoma reaches a more advanced stage, disease severity becomes the principal determinant of healthcare expenditure. The seemingly reduced overall costs observed among stage IV patients with prevalent tumors should be interpreted with caution; it appears that these patients receive less intensive care. These results have important implications for healthcare planning. As the population of cancer survivors continues to increase, an increasing proportion of patients diagnosed with melanoma will have a history of previous malignancy. Our findings indicate that this subgroup represents a distinct population with greater healthcare needs, particularly in early-stage disease, where previous cancer history substantially increases resource consumption despite the favorable prognosis of melanoma itself. Health systems should therefore consider previous malignancy status when estimating the economic burden of melanoma and when designing follow-up pathways aimed at optimizing resource allocation. Patients with concurrent malignancies represent a vulnerable and economically complex subgroup who may benefit from integrated, multidisciplinary survivorship care that balances CM-specific treatment benefits with overall health status and competing risks. Future research should explore tailored follow-up pathways, optimize treatment decision-making for multimorbid patients, and examine long-term economic implications to improve both clinical outcomes and healthcare sustainability in melanoma care.

This study’s strengths include its real-world design, which incorporates a large, population-based, high-quality cancer registry linked with administrative data. This provides sample data with greater external validity and statistical power than center-specific studies. The present study’s findings contribute to our understanding of the background of different prevalent cancers in melanoma patients.

The study also acknowledges certain limitations. Our retrospective investigation is confined to a single region in northern Italy, where healthcare services are provided under a publicly funded system. The results may not be representative of other regions, or the global population and caution should be exercised when applying these findings to different healthcare contexts. Furthermore, the present results focus on direct healthcare costs obtained from hospital and administrative data; therefore, indirect costs were not included in the analysis.

### 4.1 Conclusions

This study provides population-based evidence that the presence of prevalent primary tumors at the time of melanoma diagnosis is associated with significantly worse overall survival, but not with melanoma-specific survival. Additionally, patients with prevalent tumors incurred higher overall and CM-specific costs.

## Data Availability

The datasets presented in this study can be found in online repositories. The names of the repository/repositories and accession number(s) can be found below: https://doi.org/10.6084/m9.figshare.31915941 Figshare repository.
